# Prostate-specific antigen testing and opportunistic prostate cancer screening: a cohort study in England, 1998–2017

**DOI:** 10.3399/bjgp20X713957

**Published:** 2021-01-12

**Authors:** Ashley Kieran Clift, Carol AC Coupland, Julia Hippisley-Cox

**Affiliations:** Professor of Clinical Epidemiology and Primary Care Nuffield Department of Primary Care Health Sciences, University of Oxford, Oxford.; School of Medicine, University of Nottingham, Nottingham.; Professor of Clinical Epidemiology and Primary Care Nuffield Department of Primary Care Health Sciences, University of Oxford, Oxford.

**Keywords:** cohort studies, primary health care, prostate cancer, prostate-specific antigen, screening

## Abstract

**Background:**

Prostate cancer is a leading cause of cancer- related death. Interpreting the results from trials of screening with prostate-specific antigen (PSA) is complex in terms of defining optimal prostate cancer screening policy.

**Aim:**

To assess the rates of, and factors associated with, the uptake of PSA testing and opportunistic screening (that is, a PSA test in the absence of any symptoms) in England between 1998 and 2017, and to estimate the likely rates of pre-randomisation screening and contamination (that is, unscheduled screening in the ‘control’ arm) of the UK-based Cluster Randomised Trial of PSA Testing for Prostate Cancer (CAP).

**Design and setting:**

Open cohort study of men in England aged 40–75 years at cohort entry (1998–2017), undertaken using the QResearch database.

**Method:**

Eligible men were followed for up to 19 years. Rates of PSA testing and opportunistic PSA screening were calculated; Cox regression was used to estimate associations.

**Results:**

The cohort comprised 2 808 477 men, of whom 631 426 had a total of 1 720 855 PSA tests. The authors identified that 410 724 men had opportunistic PSA screening. Cumulative proportions of uptake of opportunistic screening in the cohort were 9.96% at 5 years’, 22.71% at 10 years’, and 44.13% at 19 years’ follow-up. The potential rate of contamination in the CAP control arm was estimated at 24.50%.

**Conclusion:**

A substantial number of men in England opt in to opportunistic prostate cancer screening, despite uncertainty regarding its efficacy and harms. The rate of opportunistic prostate cancer screening in the population is likely to have contaminated the CAP trial, making it difficult to interpret the results.

## INTRODUCTION

Prostate cancer is a common malignancy and a common cause of cancer-related death in men across many healthcare systems globally.^1^^–^^5^ The role of screening for prostate cancer using prostate-specific antigen (PSA) testing in asymptomatic men has been evaluated in several randomised controlled trials (RCTs),^6^^–^^10^ but results are difficult to assimilate into a cohesive narrative regarding the risks and benefits of such screening. Several RCTs also have major methodological limitations, mostly due to contamination of the control groups — that is, men randomised to a non-screening arm who then undergo opportunistic screening tests.^11^^–^^13^

The largest trial to date, the Cluster Randomised Trial of PSA Testing for Prostate Cancer (CAP), found no statistically significant difference in prostate cancer-specific mortality between controls and those in the study arm who were offered low-intensity screening with a one-off PSA test.^9^ However, the degree of contamination in the control arm was not empirically assessed; this is particularly important, given that the intervention arm’s screening uptake was only 36%. If the control arm of the CAP trial was substantially contaminated, this could have biased the results, potentially masking the true effect of the study intervention.

Although no formal screening programme exists in the UK, men may opt into screening as they can access PSA testing via shared decision making with their GP. The uptake of PSA testing and trends therein for some countries, particularly the US, has been relatively well characterised in several studies;^14^^–^^16^ the uptake of PSA testing, in general, in the UK has been studied in several cross-sectional^17^^–^^19^ and longitudinal studies,^20^ but these reports are limited as they tend to have narrow timeframes of interest, divergent geographical foci, restricted evaluation of sociodemographic associations with PSA testing uptake, and/or do not attempt to distinguish between men having PSA tests to investigate symptoms indicative of prostatic hypertrophy and men who are asymptomatic and having PSA tests to detect early-stage cancer.

In this study, a comprehensive analysis of the uptake of PSA testing across England was undertaken using linked datasets, and the uptake of opportunistic PSA screening in men who were asymptomatic was analysed. The primary objective was to quantify the cumulative incidence of PSA testing and opportunistic PSA screening between 1998 and 2017; the secondary objectives comprised identifying associations between PSA testing/opportunistic PSA screening rates and sociodemographic and protective/risk factors for prostate cancer.^21^^,^^22^ Furthermore, population-based estimates of the potential pre-randomisation testing and control-arm contamination of the CAP trial, based on time-period- and age-group-restricted analyses of the cohort, were derived.

**Table:** How this fits in

Men in the UK are generally regarded as having a low uptake of prostate cancer screening, but the largest ever screening trial, the Cluster Randomised Trial of PSA [prostate-specific antigen] Testing for Prostate Cancer, was based in the UK. In this observational study, to the authors’ knowledge, the largest and most comprehensive assessment of opportunistic prostate cancer screening behaviours in England was performed in a cohort study of >2.8 million men. It was found that a sizeable proportion of men opt in to opportunistic PSA screening every year The results suggest that this ‘screening on demand’ may have significant implications. For example, nuanced interpretation of trials in UK men may be needed, and rates of opportunistic screening may contextualise observed trends in prostate cancer diagnosis and outcomes.

## METHOD

A cohort study of men identified from the QResearch database (version 42) was undertaken. The database has accrued anonymised data on approximately 28 million patients over 25 years from 1457 UK-based general practices that implement the Egton Medical Information Systems (EMIS) computer system; it is representative of the UK population.^22^^–^^24^ The extracted dataset (2.8 million patients) had full linkage to: the Office for National Statistics’ mortality records; Hospital Episode Statistics records for dates of prostate biopsies and operations/treatment; and Public Health England cancer registration data regarding the date of a prostate cancer diagnosis.

Individual practices were eligible for inclusion if they contributed data within the study period of interest — namely, between 1 January 1998 and 31 March 2017. Men aged 40–75 years at study entry were eligible for inclusion. Entry to the open cohort was permitted when:

- men had no previous PSA test and no previous prostate disease diagnosis/investigation/treatment;
- men had at least 12 months’ registration with their practice;
- men had had their 40th birthday; and
- the practice had been using EMIS for at least 12 months.

The latest of these dates was defined as the cohort entry date. Men were excluded if they had:

- a pre-existing diagnosis of prostate cancer;
- prostatic hypertrophy;
- previously had a prostatectomy;
- previously had anti-androgen therapy; or
- other prostate-directed surgery or biopsy recorded prior to the cohort entry date recorded on any of the linked data sources.

Data regarding multiple risk factors for prostate cancer (including age, ethnicity, family history of prostate cancer, smoking status, and comorbidities) were extracted, as well as records of PSA tests and dates, Read codes for urinary symptoms (and dates), and cause of death.^22^

Given the age groups eligible for inclusion and the years in which the trial was conducted, the same cohort of men was used for the main analyses; however, a subgroup of the cohort was used for analyses relevant to the CAP trial. This sub-cohort comprised men aged 50–69 years.

Study outcomes were PSA testing (any man having a PSA test) and opportunistic PSA screening, which was defined as a PSA test being performed on men with no Read-coded urinary symptoms recorded at any time prior to the test.

### Statistical analysis

Kaplan–Meier failure functions were used to estimate the cumulative risks of having at least one PSA test and at least one opportunistic screening PSA test during follow-up (1998–2017). For the former, follow-up was calculated from the cohort entry date to the date of the first PSA test or censoring (cohort exit due to earliest of study end date, death, transfer out of practice, or diagnosis of prostate cancer). For the latter, follow-up was calculated from entry to censoring (cohort exit due to study end date, death, leaving the practice, or having a PSA test in the context of urinary symptoms suggestive of prostate pathology as per the aforementioned Read codes). There was no upper age limit for censoring.

Failure functions for both endpoints were stratified by ethnicity, body mass index (BMI) categories, Townsend deprivation quintile, smoking status, diabetes, geographical region, bipolar disorder or schizophrenia, and recorded family history of prostate cancer. Log-rank tests were used to identify significant differences between strata of covariates. Person-year methodology was used to calculate age-group-specific rates of PSA testing and opportunistic screening.

Trends in annual rates of men starting PSA testing and opportunistic screening between 1998 and 2016 (complete years) were analysed by calculating the percentage of men, entered in each calendar year, who were undergoing their first PSA test. Temporal trends in testing and opportunistic screening were assessed using annual percentage changes (APC) calculated using Joinpoint regression analyses.^25^^,^^26^ The parametric method was used to calculate APC confidence intervals (CIs).

Pre-randomisation opportunistic screening was estimated in the 3 years prior to the CAP trial by identifying a subgroup of males aged 50–69 years in the study cohort between 1 January 1998 and 31 December 2000; Kaplan–Meier failure functions were utilised. The potential rate of contamination of the control arm of the CAP trial was estimated by calculating the cumulative risks of having a PSA test deemed to be for opportunistic screening in men aged 50–69 years who entered the cohort between 1 January 2001 and 31 March 2016. The dates and age groups for these sub-analyses correspond to the timeframes and inclusion criteria of the CAP trial respectively. Follow-up and censoring were as outlined above.

Cox proportional-hazards models were utilised in the whole study cohort to ascertain independent predictors of undergoing a PSA test and opportunistic PSA screening (as complete case analyses). Results are reported as adjusted hazard ratios (HRs), with 95% CIs and *P*-values. The proportional-hazards assumption was assessed. All statistically significant risk factors (*P*<0.01) identified in univariate analyses were included in a single multivariable Cox regression model.

Joinpoint regression analyses utilised Joinpoint Regression Program 4.6.0; all other statistical analyses were executed using Stata (version 15.1). The significance level was set at *P*<0.01 to account for the large sample size and multiple testing.

## RESULTS

### Study population

The cohort that was initially extracted comprised 3 211 276 men aged 40–74 years from a total of 1457 general practices in England, which represented 19.60% of all the 7435 practices in England as of June 2017. After exclusions, the final study cohort comprised 2 808 477 men who had not had a previous PSA test (total follow-up: 21 569 176 person-years). Median follow-up was 5.9 years (interquartile range [IQR] 2.2–13.3 years), with a maximum of 19 years. The sociodemographic characteristics of the study cohort are summarised in Table 1; in brief, 54.75% were white (36.66% had no recorded ethnicity) and 0.27% had a recorded family history of prostate cancer.

**Table 1.** Sociodemographic characteristics of the final study cohort (N = 2 808 477)

**Characteristic**	***n* (%)**
Ethnicity	
White	1 537 660 (54.75)
Indian	44 693 (1.59)
Pakistani	25 229 (0.90)
Bangladeshi	18 487 (0.66)
Other Asian	27 600 (0.98)
Caribbean	24 835 (0.88)
Black African	42 405 (1.51)
Chinese	9785 (0.35)
Other	48 299 (1.72)
Not recorded	1 029 484 (36.66)

Geographical region	
East Midlands	146 179 (5.20)
East of England	182 864 (6.51)
London	584 489 (20.81)
North East	101 938 (3.63)
North West	445 295 (15.86)
South Central	365 111 (13.00)
South East	230 681 (8.21)
South West	312 760 (11.14)
West Midlands	298 113 (10.61)
Yorkshire & Humber	141 047 (5.02)

BMI category^a^	
Underweight (<18.5 kg/m^2^)	22 770 (0.98)
Healthy weight (18.5–24.9 kg/m^2^)	731 913 (31.45)
Overweight (25.0–29.9 kg/m^2^)	1 015 095 (43.62)
Obese (30.0–39.9 kg/m^2^)	521 124 (22.39)
Severely obese (≥40.0 kg/m^2^)	36 102 (1.55)

Deprivation quintile^b^	
1 (most affluent)	548 305 (19.58)
2	550 816 (19.67)
3	558 866 (19.96)
4	565 215 (20.19)
5 (most deprived)	576 657 (20.60)

Smoking status^c^	
Non-smoker	1 212 254 (47.00)
Ex-smoker	633 556 (24.57)
Light smoker (1–9/day)	391 036 (15.16)
Moderate smoker (10–19/day)	170 700 (6.62)
Heavy smoker (≥20/day)	171 544 (6.65)

Diabetic status	
No diabetes	2 482 092 (88.38)
Type 1 diabetes	10 070 (0.36)
Type 2 diabetes	316 315 (11.26)

Family history of prostate cancer	
Yes	7641 (0.27)
No	2 800 836 (99.73)

Diagnosis of bipolar disorder or schizophrenia	
Yes	31 717 (1.13)
No	2 776 760 (98.87)

a n *= 2 327 004 (82.9% of cohort).*

b n *= 2 799 859 (99.6% of cohort).*

c n *= 2 579 090 (91.8% of cohort). BMI = body mass index.*

During follow-up, there were 50 791 diagnoses of benign prostatic hypertrophy (1.81% of the total patient cohort), 52 811 diagnoses of prostate cancer (1.88% of the total patient cohort), and 3115 deaths from prostate cancer (0.11% of the total patient cohort).

### PSA testing

In total, 631 426 men (22.48%) had at least one PSA test during the follow-up period (Table 2) (total tests = 1 720 855). The estimated cumulative risks of men having at least one PSA test were 2.28% at 1 year’s follow-up (95% CI = 2.23 to 2.32), 13.36% at 5 years’ follow-up (95% CI = 13.32 to 13.41), 29.71% at 10 years’ follow-up (95% CI = 29.64 to 29.79), and 55.25% (95% CI = 55.12 to 55.38) at 19 years’ follow-up (data not shown). There was a clear association between increasing age and higher rates of first PSA testing (Table 2).

**Table 2.** Age-specific rates per 1000 person-years in men undertaking their first PSA test for any indication and in men undergoing opportunistic screening

**Age group**	**PSA testing**			**Opportunistic PSA screening**		
	**Having first PSA test (any indication), *n***	**PSA testing rate per 1000 person-years**	**95% CI**	**Having first screening test, *n***	**Screening rate per 1000 person-years**	**95% CI**
40–49 years	51 883	11.86	11.76 to 11.96	36 564	8.36	8.27 to 8.44
50–59 years	203 023	31.05	30.92 to 31.19	139 656	21.36	21.25 to 21.47
60–69 years	225 227	51.33	51.33 to 51.54	143 248	32.65	32.48 to 32.82
70–79 years	129 470	61.39	61.39 to 61.73	77 762	36.87	36.61 to 37.13
80–89 years	21 546	68.65	67.74 to 69.58	13 347	42.53	41.82 to 43.26
90–100 years	277	63.25	56.22 to 71.15	147	39.73	34.24 to 46.10
Overall	631 426	35.62	35.53 to 35.79	410 724	23.17	23.10 to 23.24

CI = confidence interval. PSA = prostate-specific antigen.

**Figure 1.**
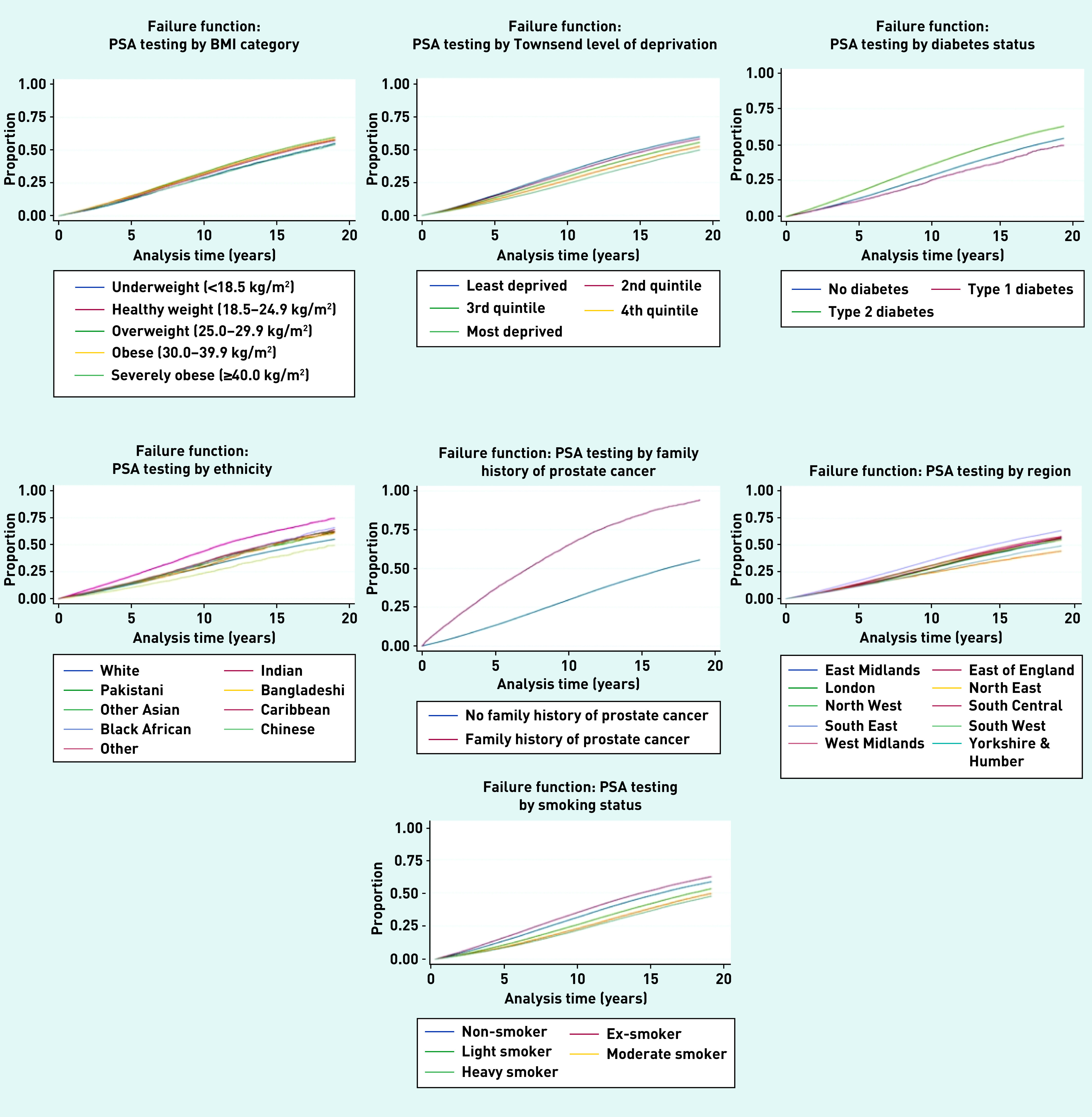
***Kaplan–Meier failure functions for factors associated with PSA testing uptake.*** ***BMI = body mass index. PSA = prostate-specific antigen.***

The cumulative risks of PSA testing when stratified by ethnicity, BMI, Townsend deprivation level, smoking status, diabetes status, geographical region, and family history of prostate cancer are shown in Figure 1. In univariate analyses, there were statistically significant differences in the cumulative risk of PSA testing when stratified (all *P*<0.0001, log-rank test). The adjusted HRs of multivariable analyses of associations with PSA testing uptake are given in Table 3.

**Table 3.** Cox regression analyses for PSA testing uptake and opportunistic screening uptake in members of the study cohort for whom data were complete (*n* = 2 305 998)^a^

	**PSA testing**			**Opportunistic PSA screening**		

**Characteristic**	**Adjusted HR^a^**	**95% CI**	***P*-value**	**Adjusted HR**	**95% CI**	***P*-value**
Age (per 1-year increase)	1.04	1.04 to 1.04	<0.0001	1.04	1.04 to 1.04	<0.0001

Townsend deprivation quintile						
1 (most affluent)	1.00			1.00		
2	0.95	0.94 to 0.96	<0.0001	0.94	0.94 to 0.95	<0.0001
3	0.88	0.88 to 0.89	<0.0001	0.87	0.86 to 0.88	<0.0001
4	0.82	0.81 to 0.82	<0.0001	0.79	0.78 to 0.80	<0.0001
5 (most deprived)	0.74	0.73 to 0.74	<0.0001	0.70	0.69 to 0.71	<0.0001

Ethnicity						
White	1.00			1.00		
Indian	1.14	1.12 to 1.17	<0.0001	1.16	1.13 to 1.18	<0.0001
Pakistani	1.20	1.16 to 1.23	<0.0001	1.12	1.08 to 1.17	<0.0001
Bangladeshi	1.17	1.13 to 1.21	<0.0001	0.99	0.94 to 1.05	0.729
Other Asian	1.15	1.12 to 1.19	<0.0001	1.11	1.07 to 1.15	<0.0001
Caribbean	1.60	1.57 to 1.64	<0.0001	1.55	1.51 to 1.60	<0.0001
Black African	1.44	1.40 to 1.48	<0.0001	1.30	1.26 to 1.35	<0.0001
Chinese	0.84	0.79 to 0.86	<0.0001	0.80	0.75 to 0.86	<0.0001
Other	1.33	1.26 to 1.40	<0.0001	1.28	1.24 to 1.32	<0.0001
Not recorded	0.75	0.74 to 0.75	<0.0001	0.76	0.75 to 0.76	<0.0001

Smoking status						
Non-smoker	1.00			1.00		
Ex-smoker	1.01	1.01 to 1.02	0.003	0.99	0.98 to 0.99	<0.0001
Light smoker	0.87	0.86 to 0.88	<0.0001	0.84	0.83 to 0.85	<0.0001
Moderate smoker	0.81	0.80 to 0.82	<0.0001	0.78	0.77 to 0.79	<0.0001
Heavy smoker	0.80	0.79 to 0.81	<0.0001	0.76	0.75 to 0.77	<0.0001

Family history of prostate cancer						
No	1.00			1.00		
Yes	3.10	3.00 to 3.19	<0.0001	3.47	3.35 to 3.60	<0.0001

Diabetes status						
No diabetes	1.00			1.00		
Type 1 diabetes	0.87	0.83 to 0.92	<0.0001	0.47	0.43 to 0.51	<0.0001
Type 2 diabetes	0.98	0.97 to 0.99	<0.0001	0.83	0.82 to 0.84	<0.0001

BMI category						
Underweight (<18.5 kg/m^2^)	1.00			1.00		
Healthy weight (18.5–24.9 kg/m^2^)	1.09	1.05 to 1.12	<0.0001	1.03	0.99 to 1.06	0.153
Overweight (25.0–29.9 kg/m^2^)	1.15	1.12 to 1.18	<0.0001	1.07	1.01 to 1.11	<0.0001
Obese (3.00–39.9 kg/m^2^)	1.15	1.12 to 1.19	<0.0001	1.04	1.01 to 1.08	0.019
Severely obese (≥40 kg/m^2^)	1.12	1.08 to 1.16	<0.0001	0.98	0.93 to 1.02	0.276

Geographical region						
East Midlands	1.00			1.00		
East of England	1.04	1.02 to 1.05	<0.0001	1.07	1.05 to 1.09	<0.0001
London	1.25	1.23 to 1.27	<0.0001	1.34	1.32 to 1.37	<0.0001
North East	0.85	0.83 to 0.86	<0.0001	0.86	0.84 to 0.87	<0.0001
North West	1.12	1.10 to 1.13	<0.0001	1.12	1.11 to 1.14	<0.0001
South Central	1.14	1.13 to 1.16	<0.0001	1.21	1.19 to 1.23	<0.0001
South East	1.41	1.39 to 1.43	<0.0001	1.46	1.44 to 1.49	<0.0001
South West	1.11	1.07 to 1.10	<0.0001	1.12	1.10 to 1.14	<0.0001
West Midlands	1.15	1.09 to 1.16	<0.0001	1.20	1.18 to 1.22	<0.0001
Yorkshire & Humber	0.91	0.89 to 0.93	<0.0001	0.90	0.88 to 0.91	<0.0001

a All significant risk factors identified in univariate analyses were included in a single Cox regression model, which is adjusted for all the other variables presented in the above table. BMI = body mass index. CI = confidence interval. HR = hazards ratio. PSA = prostate-specific antigen.

For 1998–2016, annual rates of first PSA testing ranged between 0.76% and 4.36%. Joinpoint regression analyses demonstrated statistically significant changes in the trends of PSA testing uptake: between 1998 and 2001, the APC was +41.71% (95% CI = 26.82 to 58.44, *P*<0.01); between 2001 and 2004, it was +14.83% (95% CI = 1.52 to 29.87, *P*<0.01); between 2004 and 2009, it was +5.23% (95% CI = 1.82 to 8.85, *P*<0.01); and between 2009 and 2016, it was −2.24% (95% CI = −3.61 to −0.82, *P*<0.01).

### Opportunistic PSA screening

The authors identified 410 724 men who were deemed as having undergone opportunistic screening for prostate cancer (Table 2), representing 14.62% of all men in the study cohort; as such, 65.04% of all first PSA tests in the study period [410 724 out of 631 426] were deemed to be for screening.

**Figure 2.**
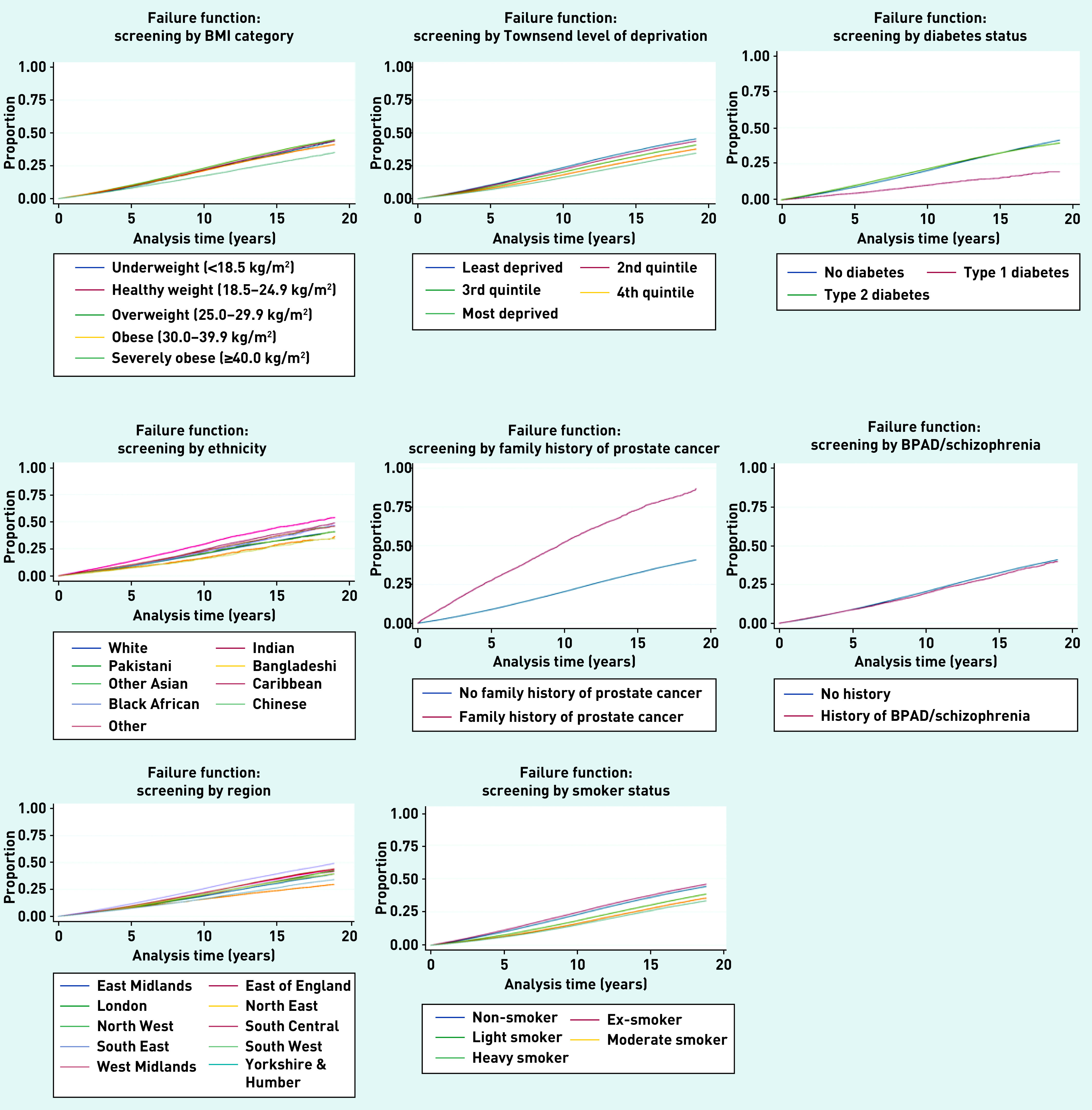
***Kaplan–Meier failure functions for factors associated with opportunistic prostate cancer screening uptake.*** ***BMI = body mass index. BPAD = bipolar affective disorder.***

The estimated cumulative risks of men undergoing at least one opportunistic screening test were 1.67% (95% CI = 1.66 to 1.69) at 1 year’s follow-up, 9.96% (95% CI = 9.92 to 10.01) at 5 years’ follow-up, 22.70% (95% CI = 22.62 to 22.77) at 10 years’ follow-up, and 44.13% (95% CI = 43.99 to 44.27) at 19 years’ follow-up. The rates of opportunistic PSA testing increased with increasing age (Table 2). The cumulative risks of undergoing a screening PSA test were statistically significantly associated with age, ethnicity (highest in Caribbean males), smoking status, positive diabetic status, geographical region, and positive family history of prostate cancer in univariate analysis (all *P*<0.0001, log-rank test, Figure 2). Table 3 demonstrates the adjusted HRs in multivariable analyses of associations with opportunistic screening uptake. There were significant associations with deprivation (inverse association), positive family history of prostate cancer, and increasing age. For example, men with a positive recorded family history of prostate cancer were over three times as likely to undergo an opportunistic screening test (HR 3.47, 95% CI = 3.35 to 3.50).

Annual percentages for men starting opportunistic prostate screening ranged between 0.46% and 2.87%. In Joinpoint analyses, the APC in screening uptake between 1998 and 2004 was +24.73% [95% CI = 15.71 to 24.46, *P*<0.01); between 2004 and 2014, it was 2.14% [95% CI = −0.32 to 4.54%], *P*>0.05). Between 2014 and 2016, there was a statistically significant decline in screening uptake, with an APC of −29.41% (95% CI = −49.91 to −0.54%, *P*<0.01). Throughout this whole period of interest, the average APC was +4.73% [95% CI = 0.41 to 9.24%].

### Estimates of potential contamination

In total, 585 166 men aged 50–69 years were identified between 1 January 1998 and 31 December 2000 (3 years preceding the CAP trial start date in 2001); of these, 13 580 (2.32%) underwent at least one PSA screening test during that period (pre-randomisation screening estimate). To estimate control-arm contamination, the authors identified men aged 50–69 years during the period of the CAP trial (2001–2016) — that is, those men who may have been eligible for inclusion. Of the 848 959 men identified, 208 041 (24.50%) had undergone at least one PSA screening test during the trial period (2001–2016) — the calculated cumulative risk of undergoing screening was 10% at 5 years’ follow-up, 23% at 10 years’ follow-up, and 36% at 15 years’ follow-up (2001–2016).

The influence of the CAP trial on the authors’ CAP-focused results — the study period was intentionally identical and some men in the cohort for the study presented here may have been included in the CAP trial. Such influence, however, should be minimal as, although 67 313 men in the CAP trial arm underwent screening,^9^ the authors identified 208 041 men aged 50–69 years who had undergone at least one screening test during the CAP trial’s follow-up period. Assuming the almost impossible scenario that *all* screened men from the CAP trial (QResearch does not cover Cardiff) were included, the estimate of opportunistic screening in the remainder is 16.58% (140 728/848 959). Even in this overestimation of the worst-case scenario, a contamination rate of close to 20% can be calculated. The CAP trial power calculations assumed that less than 20% of the control arm underwent screening.

## DISCUSSION

### Summary

To the authors’ knowledge, this is not only the largest study ever to report on the rates of PSA testing and opportunistic PSA screening in the UK population, but also the most comprehensive in terms of time period, geographical coverage, and examined risk factors. The potential rate of opportunistic prostate cancer screening in the general population of men aged ≥40 makes data from the CAP trial^9^ complex to interpret. However, increased rates of opportunistic screening were significantly associated with black African and Caribbean ethnicity, increasing age, increased affluence, and family history of prostate cancer. There were also reduced rates in men with diabetes and smokers, as well as regional variation.

### Strengths and limitations

The major strength of this intentionally contemporaneous study is the use of the QResearch database. The very large representative cohort of >2.8 million men from across England had high-quality, accurately coded, individual-level data^27^ with protracted follow-up and a low risk of bias related to selection, and recall. It also had linkages to enable the optimal ascertainment of interventions, diagnoses, deaths, and laboratory investigation results across the healthcare network.^28^^,^^29^

Limitations include the extent to which urinary tract symptoms have been recorded by GPs. This was mitigated to the best of the authors’ abilities by extracting data based on >100 Read codes, which indicate a comprehensive range of urinary tract symptoms. Other limitations of the study include information bias and missing data (such as for ethnicity).

### Comparison with existing literature

Several studies have examined the rates of PSA testing in British men;^17^^–^^21^^,^^30^ however, these comprise study cohorts smaller than those in the study presented here,^20^ have focused purely on single geographical regions,^17^ or have limited time frames.^18^^,^^19^^,^^21^^,^^30^ Other studies have identified similar associations between PSA testing and age, ethnicity, level of deprivation, and geographical region,^19^^,^^20^^,^^30^ but none has examined as extensive a panel of covariates as the study presented here, or comprehensively assessed the associations with opportunistic prostate cancer screening.

Screening uptake was statistically significantly associated with previously established risk factors for prostate cancer diagnosis, including ethnicity and positive family history of prostate cancer.^22^ As such, the status quo of informal, opportunistic screening may be an inadvertent manifestation of a patient-led, risk-adapted strategy or one guided by some GPs.

### Implications for practice

Men seeking PSA screening in general practice may not be uncommon, which gives a mandate for a deep awareness among clinicians of the benefits and harms of PSA screening, which is sometimes limited.^19^^,^^31^^,^^32^ Predictors of screening uptake may be useful for the design of future screening strategies, and the data presented here may help give some context to findings of other studies regarding trends in prostate cancer incidence, stage at diagnosis, treatments, and mortality in the UK.

The authors have identified factors rendering interpretation of the largest ever prostate cancer screening trial complex. Restricting the study focus to a contemporaneous sub-cohort matched by age range, it was estimated that 23% of such men in England would have had a screening test by 10 years’ follow-up; this would reduce the trial’s power to detect a difference in prostate cancer-specific mortality between screened and ‘non-screened’ arms (the power calculation has assumed a contamination rate of <20%). Statistical analyses adjusting for contamination and screening non-compliance^33^ in the CAP trial may be of great interest for screening policy, as has been done for the Prostate, Lung, Colorectal and Ovarian (PLCO) trial results,^12^^,^^34^^,^^35^ provided that they are clearly explained and robustly developed.^36^

The results presented here suggest limited plausibility of deriving clear conclusions from trials of PSA screening. The notional conclusion that one-off PSA screening is not efficacious may be over-simplistic given the likely extent of contamination, possibly incorrect, or poorly reflective of a complex situation requiring nuanced interpretation. The CAP trial and the authors’ study were conducted in a population generally regarded as having a low uptake of opportunistic screening. Given the results of the authors’ study, it appears that, even in these settings, contamination of control arms may always occur, regardless of the geographical location and design of any PSA screening trial. This may bemire evidence-based practice. In conjunction with the low probability of new trials examining PSA alone as a screening modality, policymakers, researchers, clinicians, and patients should accept that we have entered a challenging ‘post-trial’ world.
